# Control of electron-electron interaction in graphene by proximity screening

**DOI:** 10.1038/s41467-020-15829-1

**Published:** 2020-05-11

**Authors:** M. Kim, S. G. Xu, A. I. Berdyugin, A. Principi, S. Slizovskiy, N. Xin, P. Kumaravadivel, W. Kuang, M. Hamer, R. Krishna Kumar, R. V. Gorbachev, K. Watanabe, T. Taniguchi, I. V. Grigorieva, V. I. Fal’ko, M. Polini, A. K. Geim

**Affiliations:** 1 School of Physics and Astronomy, University of Manchester, Manchester, M13 9PL UK; 2 National Graphene Institute, University of Manchester, Manchester, M13 9PL UK; 3 Saint-Petersburg INP, Gatchina, 188300 Russia; 4 National Institute for Materials Science, Tsukuba, 305-0044 Japan; 5 Dipartimento di Fisica dell’Università di Pisa, Largo Bruno Pontecorvo 3, 56127 Pisa, Italy; 6 Istituto Italiano di Tecnologia, Graphene Labs, Via Morego 30, 16163 Genova, Italy

**Keywords:** Nanoscience and technology, Physics

## Abstract

Electron-electron interactions play a critical role in many condensed matter phenomena, and it is tempting to find a way to control them by changing the interactions’ strength. One possible approach is to place a studied system in proximity of a metal, which induces additional screening and hence suppresses electron interactions. Here, using devices with atomically-thin gate dielectrics and atomically-flat metallic gates, we measure the electron-electron scattering length in graphene and report qualitative deviations from the standard behavior. The changes induced by screening become important only at gate dielectric thicknesses of a few nm, much smaller than a typical separation between electrons. Our theoretical analysis agrees well with the scattering rates extracted from measurements of electron viscosity in monolayer graphene and of umklapp electron-electron scattering in graphene superlattices. The results provide a guidance for future attempts to achieve proximity screening of many-body phenomena in two-dimensional systems.

## Abstract (web summary)

Experimental control of electron-electron interactions in materials is challenging. Here, the authors control the interactions by proximity screening with gate dielectrics of nanometer thickness, revealing qualitative changes in concentration and temperature dependences, and validating their analysis using electron hydrodynamics and umklapp scattering approaches.

## Introduction

Electrostatic screening by conducting gates has previously been employed to suppress charge inhomogeneity in graphene^1–3^, alter its plasmon spectra^4,5^, and renormalize electronic spectra of monolayer semiconductors^6,7^. Elementary electrostatics tells us that the electron charge $e$ placed at the distance $d$ from a bulk conductor leads to a dipole potential evolving as $2ed^2/r^3$ at large in-plane distances $r \gg d$, which is much weaker than the original, unscreened Coulomb potential, $e/r$. Accordingly, a metallic gate placed sufficiently close to another electronic system can alter its electron–electron (e–e) interactions. They can be parametrized by the e–e scattering length $\ell _{{\mathrm{ee}}}$. From the above considerations, one can infer that what matters most is the ratio $d/D$, where $D\approx1/\sqrt n$ is the average separation between electrons and $n$ is the carrier concentration. For a two-dimensional (2D) electron system with typical $n = 10^{12}\,{\mathrm{cm}}^{ - 2}$, $D \approx 10\,{\mathrm{nm}}$ and, therefore, the inferred gate separation $d \approx D$ is relatively easy to achieve experimentally. However, as shown below, the naïve expectations fail because of a small numerical factor $\delta$ such that e–e interactions for massless Dirac fermions are altered only if $d \le \delta D \approx0.03\,\varepsilon D$, where $\varepsilon$ is the gate dielectric’s permittivity. For typical gate dielectrics with $\varepsilon \,{<}\, 5$, the required separation falls into a $1\,{\mathrm{nm}}$ range. For massive charge carriers such as those in bilayer graphene and 2D semiconductors, even smaller (atomic scale) $d$ are necessary for efficient screening (Methods). It seems impossible to realize the required small $d$ because of, for example, inevitable surface roughness of the conducting and insulating films used for gating and electrical leakage through dielectrics of nanometer thickness.

In this communication, we achieve the extremely challenging conditions for proximity-gate screening by using van der Waals heterostructures with atomically thin dielectric layers and atomically flat gates. Measurements of viscous electron flow in graphene and umklapp scattering rates in graphene superlattices provide two independent but complementary ways to quantify the effect of proximity screening and its dependence on $d$, $n$, and temperature ($T$).

## Results

### Experimental devices and measurement setup

**Fig. 1:**
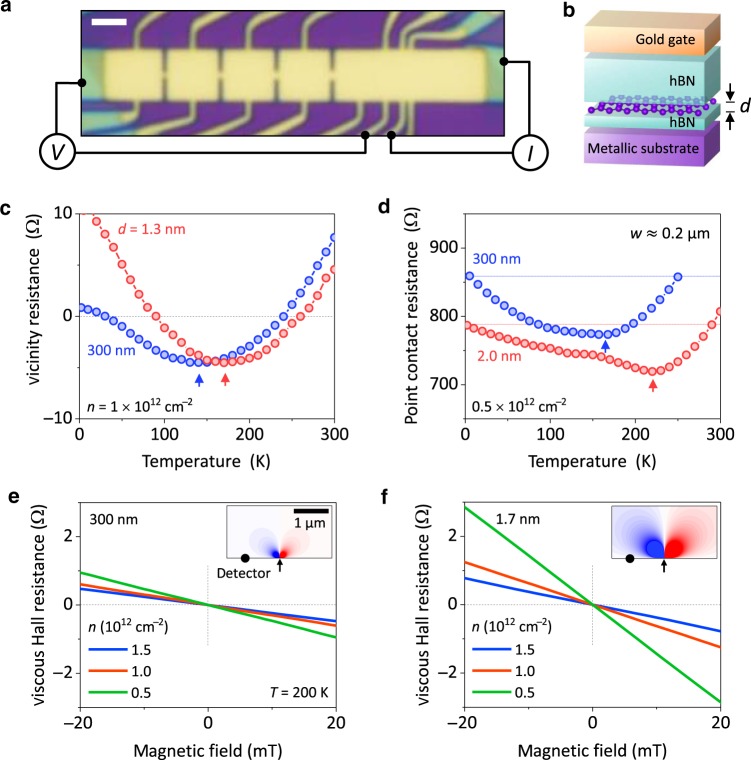
Graphene devices with proximity gating and its effect on electron hydrodynamics. **a** Optical micrograph of one of our devices with four sub-μm constrictions used for point-contact measurements and several closely spaced contacts for vicinity measurements. The wiring schematic illustrates current and voltage configurations for the latter measurements. Scale bar, 2 μm **b** Schematic side view of our heterostructures. **c** $R_{\mathrm{V}}$ as a function of $T$ for representative devices with a close graphite gate ($d \approx 1.3\,{\mathrm{nm}}$, red) and in the reference geometry ($d = 300\,{\mathrm{nm}}$, blue). The devices had similar geometry and $\mu$; same *L* = 0.5 μm. **d** $R_{{\mathrm{PC}}}(T)$ for screened and reference constrictions of the same width $w \approx 0.2\,\upmu {\mathrm{m}}$ (same color coding as in **c**). Dashed lines in **d** denote the resistance in the ballistic limit. Arrows in **c** and **d** indicate minima in $R_{\mathrm{V}}$ and $R_{{\mathrm{PC}}}$. **e**, **f** Viscous Hall effect for reference and close-gate devices ($d = 300$ and $1.7\,{\mathrm{nm}},$ respectively). The color-coded curves correspond to different $n;$ all measurement conditions and geometries were same, including $L = 1\,\upmu {\mathrm{m}}$ and $T = 200\,{\mathrm{K}}$. The insets illustrate electric potentials that appear due to a viscous electron flow (the arrow and circle indicate positions of current and voltage contacts, respectively). The calculations^17^ were carried out for the experimentally determined $\ell _{{\mathrm{ee}}} \approx 0.3$ and $0.8\,\upmu {\mathrm{m}}$ for panels **e** and **f**, respectively; $B = 10\,{\mathrm{mT}}$. Blue-to-red color scale is arbitrary but same for both panels.

Our devices were graphene monolayers encapsulated between hexagonal boron nitride (hBN) crystals whereas graphite monocrystals served as a bottom gate (Fig. 1). These heterostructures were fabricated using the standard dry-transfer procedures^1^ described in Methods. Multiterminal Hall bar devices with several point contacts and closely placed voltage probes (Fig. 1a) were then defined by electron-beam lithography and plasma etching. An extra metal gate was deposited on top of the heterostructures, which allowed us to vary $n$ without applying voltages to the bottom screening gate. This was particularly important for our case of ultra-thin dielectrics to avoid their accidental breakdown and electrical leakage. The minimum thickness $d$ for the gate dielectric (Fig. 1b) was limited to four hBN layers (i.e. ∼$1.3\,{\mathrm{nm}}$) because thinner crystals exhibited notable electron tunneling^8^. The devices typically had low-$T$ mobility $\mu$ of about $10^6\,{\mathrm{cm}}^2\,{\mathrm{V}}^{ - 1}\,{\mathrm{s}}^{ - 1}$ and highly reproducible characteristics such that, at finite $T$, their longitudinal resistivity $\rho$ was practically independent of $d$ (Supplementary Note 1; Supplementary Fig. 1). This ensured that the reported behavior of $\ell _{{\mathrm{ee}}}$ was due to changes in $d$ rather than transport characteristics. Because graphite is a semimetal with a relatively low carrier density of ∼$10^{19}\,{\mathrm{cm}}^{ - 3}$, we also crosschecked that our conclusions were independent of the gate material by using screening gates made from layered metals such as Bi_2_Sr_2_CaCu_2_O_8+*x*_ and TaS_2_ (Methods; Supplementary Fig. 2).

To demonstrate that e–e interactions can be tuned by proximity-gate screening, a reliable diagnostic tool is essential. Many quantum transport characteristics are known to be affected by the strength of e–e interactions. For example, the phase breaking length depends on it and can be measured in quantum interference experiments^9^ (other possibilities are discussed in ref. ^10^). In principle, it should be possible to use such mesoscopic physics tools to probe e–e interactions in graphene but, because of its ballistic transport at micrometer-scale distances, the approach is not easy to implement in practice and its results could be difficult to interpret. On the other hand, recent experiments have shown that graphene at finite $T$ and away from the charge neutrality point (NP) exhibits pronounced hydrodynamic effects^11–15^, which allowed measurements of the kinematic electron viscosity $\nu _0$. The extracted values of $\ell_{{\mathrm{ee}}}$, obtained by inverting the relation $\nu _0 = v_{\mathrm{F}}\ell_{{\mathrm{ee}}}/4$ derived in refs. ^16–18^ ($v_{\mathrm{F}} \approx 10^6\,{\mathrm{ms}}^{ - 1}$ is the Fermi velocity), were in quantitative agreement with theory (because viscosity stems from e–e interactions, the proportionality is not surprising and dimensionally correct). The viscosity measurements can be carried out using three complementary approaches: vicinity resistance^12,13^, point-contact geometry^14,18^, and the viscous Hall effect^15^. Below we use all three to show that $\ell _{{\mathrm{ee}}}$ changes with $d$. In a complementary approach, we demonstrate that umklapp e–e scattering in graphene superlattices^19^ is also affected by proximity-gate screening.

### Enhanced electron viscosity

First, we demonstrate the screening effect qualitatively. Figure 1c shows that the vicinity resistance $R_{\mathrm{V}}$ is notably affected if a thin gate dielectric is employed. Vicinity measurements are discussed in detail in ref. ^12^ but, briefly, an electric current is injected through a narrow contact into a wide graphene channel. The negative voltage drop arising locally from a viscous electron flow is detected using a vicinity contact at a short distance $L$ from the current-injecting contact (Fig. 1a). One can see from Fig. 1c that, as $T$ increases, $R_{\mathrm{V}}$ first decreases and then becomes negative. This indicates a transition from the ballistic transport regime (positive $R_{\mathrm{V}}$) into a regime where ballistics is strongly affected by e–e scattering^13^. The minimum in $R_{\mathrm{V}}(T)$ corresponds to the condition $\ell _{{\mathrm{ee}}} \approx L$ and indicates an onset of hydrodynamic behavior^13^. As $\ell _{{\mathrm{ee}}}$ decreases further with increasing $T$, $R_{\mathrm{V}}$ becomes less negative and eventually positive, being dominated by currents caused by electron–phonon scattering^12,13^. The dependences $R_{\mathrm{V}}(T)$ shown in Fig. 1c were measured for two similar devices at the same $L$. One had $d \approx 300\,{\mathrm{nm}}$ (conventional Si back gate) whereas the other was made using four-layer hBN as the gate dielectric. Despite the similar behavior of $R_{\mathrm{V}}(T)$, the curve for $d \approx 1.3\,{\mathrm{nm}}$ is clearly shifted to higher $T$. The shift direction indicates that the nearby gate causes an increase in $\ell _{{\mathrm{ee}}}$, which is equivalent to a reduction in electron temperature by ∼$30\,{\mathrm{K}}$. Note that for $T$ above $100\,{\mathrm{K}}$ where the hydrodynamic regime develops, electron transport in high-quality graphene is universal and insensitive to experimental details.

Similar phenomenology was observed in the point-contact geometry (Fig. 1a). Again, the $T$-dependence of the point-contact resistance $R_{{\mathrm{PC}}}$ exhibits a clear minimum due to a viscous flow^14^. The shift to higher $T$ for the device with a proximity gate (Fig. 1d) indicates an increase in $\ell _{{\mathrm{ee}}}$ for a given $T$ (also, see Supplementary Note 3). Such influence of the proximity gating was consistently observed in all our experiments. The $R_{\mathrm{V}}$ and $R_{{\mathrm{PC}}}$ dependences could also be used to extract $\ell _{{\mathrm{ee}}}(T)$ following the recipe reported in refs. ^12,14^. Unfortunately, we found that, for atomically thin gate dielectrics, detailed behavior of $R_{\mathrm{V}}$ and, to some extent, $R_{{\mathrm{PC}}}$ notably varied between different devices with nominally the same $d$. Those variations can be traced back to the fact that $R_{\mathrm{V}}$ is sensitive to current injector’s geometry^12^ whereas a viscous contribution to $R_{{\mathrm{PC}}}$ becomes smaller for close-gate devices as compared to those with thicker gate dielectrics.

In contrast to the vicinity and point-contact measurements, the viscous Hall effect^15^ was found to be very robust, yielding quantitatively same results for different devices with same $d$. Accordingly, for quantitative analysis of how $\ell _{{\mathrm{ee}}}$ depended on $d$, we focused on the latter measurements. The Hall viscosity experiments utilize the already discussed vicinity geometry (Fig. 1a) but a non-quantizing magnetic field $B$ is applied perpendicular to graphene^15^. The field leads to an asymmetry in the potential created by the viscous flow around the injection contact (insets of Fig. 1e, f). The viscous contribution asymmetric in $B$ is called the viscous Hall resistance $R_{\mathrm{A}}$ and given by^15,17^

1 $$R_{\mathrm{A}} = \rho \xi \left( {\frac{L}{{\sqrt {\nu _0\tau } }}} \right)\frac{B}{{B_0}},$$

where $\xi \left( x \right)$ is a dimensionless function^17^, $\tau$ is the transport scattering time, $B_0 = E_{\mathrm{F}}/(8|e|\nu _0)$ is a characteristic magnetic field, and $E_{\mathrm{F}}$ is the Fermi energy. Because $|\xi \left( x \right)|$ is a monotonically decreasing function of its argument for $x \,{> }\, 0$, $|R_{\mathrm{A}}|$ increases with increasing $\ell _{{\mathrm{ee}}}$ and, accordingly, devices with weaker e–e scattering should exhibit larger $|R_{\mathrm{A}}|$.

To illustrate the effect of proximity-gate screening on Hall viscosity, Fig. 1e, f plot $R_{\mathrm{A}}(B)$ for two representative devices with $d \approx 1.7$ and $300\,{\mathrm{nm}}$. The curves are taken under exactly the same conditions for several same$n$. As the two devices exhibited close $\rho$ and $\tau$ (Supplementary Fig. 1), the profound difference between Fig. 1e and f can only be attributed to different screening. The device with the thin dielectric exhibited much larger Hall viscosity than the reference device, and the effect was most pronounced at low $n$. This behavior proves again that the proximity screening suppresses e–e scattering, in agreement with the conclusions reached from the vicinity and point-contact measurements.

**Fig. 2:**
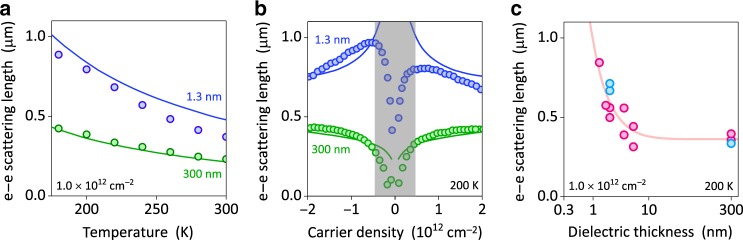
Dependence of the e-e scattering length on distance to the gate. **a** $\ell _{{\mathrm{ee}}}(T)$ extracted from Hall viscosity measurements for the given $n$. Data for a close-gate device (blue symbols) are compared with a reference (green). **b** Density dependence of $\ell _{{\mathrm{ee}}}$ at $200\,{\mathrm{K}}$ (same color coding as in **a**). The gray-shaded region indicates the regime near the NP where the single-component hydrodynamic theory is not applicable^12,13,20^ and, also, the cyclotron diameter became comparable with the width of our devices^15^. **c** $\ell _{{\mathrm{ee}}}$ as a function of $d$ for the given $n$ and $T$. Red and blue symbols: Results from Hall viscosity and point-contact measurements, respectively; shown are the average values for electron and hole doping (see panel **b** for an example of scatter due to electron–hole asymmetry). For all the panels, the solid curves are theoretical results (Supplementary Note 4).

For the known transport characteristics ($\rho$ and $\tau$), Eq. (1) allows us to convert $R_{\mathrm{A}}$ into $\ell _{{\mathrm{ee}}}$, as described in detail in ref. ^15^. Figure 2a shows examples of $\ell _{{\mathrm{ee}}}(T)$ found for close-gate and reference devices. At all $T$, the screened device displays $\ell _{{\mathrm{ee}}}$ approximately twice longer than that in the standard device of the same electronic quality. This agrees well with many-body theory (solid curves in Fig. 2; Supplementary Fig. 3). Importantly, the proximity-gate screening qualitatively changes the dependence $\ell _{{\mathrm{ee}}}(n)$ so that, away from the NP, $\ell _{{\mathrm{ee}}}$ decreases with increasing $n$ (Fig. 2b). This contrasts with monotonically increasing $\ell _{{\mathrm{ee}}}(n)$ for the reference devices, which was also reported previously^14,15^. Figure 2c summarizes our results by showing $\ell _{{\mathrm{ee}}}$ measured for more than 10 different devices at characteristic $n$ and $T$ where viscous effects become most pronounced in graphene. Despite the experimental scatter, Fig. 2c clearly shows that $\ell _{{\mathrm{ee}}}$ can be altered appreciably by using thin gate dielectrics, if $d$ is smaller than a few nm.

To explain the observed dependences of $\ell _{{\mathrm{ee}}}$ on $n$ and $d$, we carried out numerical calculations in the random phase approximation for the dynamically screened interactions^10,21,22^. The gate was modeled as a perfect conductor, and small departures from this model caused by a finite carrier density were estimated in Supplementary Note 4. The results are shown by the solid curves in Fig. 2. No fitting parameters were used, except for multiplying all the theoretical curves by the same small factor of $1.3$ (its non-Fermi-liquid origins are discussed in Supplementary Note 4). However, to gain better insight about the observed behavior, we also derived the following analytical expression:

2 $$\ell _{{\mathrm{ee}}}\approx\frac{{4\hbar v_{\mathrm{F}}E_{\mathrm{F}}}}{\pi }\frac{1}{{\left( {k_{\mathrm{B}}T} \right)^2\ln \left( {\frac{{2E_{\mathrm{F}}}}{{k_{\mathrm{B}}T}}} \right)}}\left( {\frac{{1 + 2dq_{{\mathrm{TF}}}}}{{2dq_{{\mathrm{TF}}}}}} \right)^2,$$

where $k_{\mathrm{F}} = \sqrt {\pi n}$ and $q_{{\mathrm{TF}}} = 4\alpha _{{\mathrm{ee}}}k_{\mathrm{F}}$ are the Fermi and Thomas–Fermi wavenumbers, respectively. Here, $\alpha _{{\mathrm{ee}}} \equiv e^2/({\it{\epsilon }}\hbar v_{\mathrm{F}}) \approx 2.2/\varepsilon$ is graphene’s coupling constant and $k_{\mathrm{B}}$ is the Boltzmann constant (Supplementary Note 4 discusses the case of generally anisotropic $\varepsilon$). The expression is accurate in the Fermi-liquid regime ($k_{\mathrm{B}}T \ll E_{\mathrm{F}}$), where it matches our numerical results (Supplementary Note 4). The last term in Eq. (2) appears due to the gate presence, and the key parameter describing its screening effect is $dq_{{\mathrm{TF}}}$. In the far-gate regime, $d \gg 1/q_{{\mathrm{TF}}}$, Eq. (2) reduces to the standard unscreened expression^22^. In the opposite limit, $d \ll 1/q_{{\mathrm{TF}}}$, e–e scattering is strongly reduced due to screening, and $\ell _{{\mathrm{ee}}}$ increases with decreasing both $d$ and $n$, as $1/d^2$ and approximately $1/\sqrt n$, respectively, in agreement with our experiment (Fig. 2). The latter dependence is opposite to the unscreened case, where $\ell _{{\mathrm{ee}}}$ increases as $\sqrt n$, in agreement with the results of Fig. 2b. The crossover between the far- and close-gate regimes occurs at a critical distance $d_{\mathrm{c}}$ such that $d_{\mathrm{c}} \approx 1/2q_{{\mathrm{TF}}} = 1/(8\alpha _{{\mathrm{ee}}}k_{\mathrm{F}})$, which translates into the previously introduced parameter $\delta \approx0.03\varepsilon$. For hBN with $\varepsilon \approx 3.5$ and at typical $n = 10^{12}\,{\mathrm{cm}}^{ - 2}$, we obtain $d_{\mathrm{c}} \approx 1.1\,{\mathrm{nm}}$, which explains why the gate screening becomes noticeable only for our smallest $d$ (Fig. 2c). Further information about our theoretical analysis is provided in Supplementary Note 4.

### Suppression of e–e umklapp scattering

**Fig. 3:**
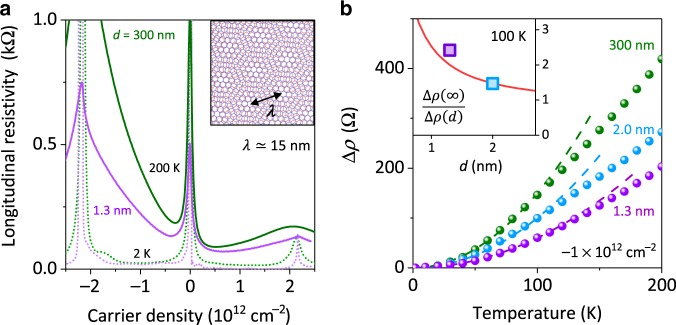
Suppression of umklapp e–e scattering in graphene superlattices by proximity-gate screening. **a** $\rho$($n$) of graphene-on-hBN superlattices for $d \approx 1.3$ and $300\,{\mathrm{nm}}$ (purple and green curves, respectively). Dotted and solid curves: $T = 2$ and $200\,{\mathrm{K}}$, respectively. Inset: Illustration of the moiré pattern arising from crystallographic alignment between graphene and hBN lattices. **b** $T$-dependent part of $\rho$ for superlattice devices with different $d$ (color-coded symbols); $n = - 1 \times 10^{12}\,{\mathrm{cm}}^{ - 2}$ so that superlattices’ first Brillouin zones are approximately half-filled with holes^25–27^. Dashed curves: Best fit to the predicted $T^2$ dependence^19^. All the devices had $\lambda \approx 15\,{\mathrm{nm}}$ and close $\rho$ at $2\,{\mathrm{K}}$. Inset: ${\mathrm{\Delta }}\rho (d)$ for the two close-gate superlattices normalized by ${\mathrm{\Delta }}\rho \left( \infty \right)$ measured for the reference (far-gate) superlattice. The color-coded symbols in the inset are taken from the main panel and valid for all $T \le 120\,{\mathrm{K}}$ because of the $T^2$ dependence. Solid curve: theory.

To check how robust our conclusions are, we have also examined the effect of gate-induced screening on umklapp e–e scattering^19^ that dominates resistivity $\rho$ of graphene-on-hBN superlattices at elevated $T$. We made several superlattice devices with the moiré periodicity $\lambda \approx 15\,{\mathrm{nm}}$, as confirmed by the periodicity of Brown–Zak oscillations^23^ and the appearance of secondary NPs^24–27^ at the expected $n$ (Fig. 3a). One of the devices was the standard Hall bar with $d = 300\,{\mathrm{nm}}$, like those reported previously^19^. The other two were same in design but had a bottom graphite gate placed at short $d$, as in the above viscosity experiments. Figure 3 shows typical $\rho (n,T)$ measured for these graphene superlattices. For $d = 300\,{\mathrm{nm}}$, the observed behavior was same as reported previously, and the $T$-dependent part ($\Delta \rho$) of graphene superlattice’s resistivity could be described quantitatively by umklapp e–e scattering^19^. It is responsible for the rapid increase of $\Delta \rho \propto T^2$ (Fig. 3b). The proximity-gate screening notably suppressed $\Delta \rho (T)$, by a factor $> 2$ for $d \approx 1.3\,{\mathrm{nm}}$. Our theoretical analysis (Supplementary Note 5) shows that ${\mathrm{\Delta }}\rho$ for the close-gate devices should exhibit the same $T$ dependence (${\propto} T^2$) but with a reduced absolute value. The umklapp e–e scattering length, $\ell _{{\mathrm{ee}}}^{\mathrm{U}}$, is governed by distinctive processes with a momentum transfer of ${\sim} \hbar g$ where $g = \frac{{4\pi }}{{\sqrt 3 }}\lambda ^{ - 1}$ is the superlattice reciprocal vector. As shown in Supplementary Note 5, proximity screening for $\ell _{{\mathrm{ee}}}^{\mathrm{U}}$ becomes important if $d \,{<}\, 0.1\lambda$, which means that few-nm-thick gate dielectrics are essential to observe the screening effect, similarly to the case of hydrodynamic transport. It is convenient to quantify the changes in umklapp scattering by the dimensionless ratio, $\Delta \rho \left( \infty \right)/\Delta \rho \left( d \right)\approx\ell _{{\mathrm{ee}}}^{\mathrm{U}}(d)/\ell _{{\mathrm{ee}}}^{\mathrm{U}}(\infty )$. The results are plotted in the inset of Fig. 3b and show good agreement with theory (for details, see Supplementary Note 5 and Supplementary Fig. 4).

## Discussion

Electron–electron scattering in monolayer graphene at finite $n$ can strongly be suppressed if a metallic gate is placed at $d$ of about $1\,{\mathrm{nm}}$. This “close-gate” regime has become accessible due to the use of van der Waals assembly that allows atomically sharp interfaces and ultra-thin dielectrics. It is tempting to exploit the outlined strategy to assess interaction phenomena near the NP where low $n$ allow the condition $d \ll 1/\sqrt n$ to be satisfied easier but interpretation of some previous observations had proven difficult. Other interesting candidates for proximity screening are exotic phenomena driven by strong correlations (e.g., various many-body phases in twisted bilayer graphene^28–30^) and, especially, interaction effects governed by lengths longer than $\ell _{{\mathrm{ee}}}$. The experimental challenge to reach the close-gate regime can partially be mitigated by using high-$\varepsilon$ dielectrics.

## Methods

### Device fabrication

Our heterostructures were assembled using stamps made from polypropylene carbonate (PPC) as a sacrificial polymer placed on polydimethylsiloxane (PDMS). Such polymer stamps were used to pick up exfoliated thin crystals in the following sequence: top hBN (typically thicker than $30\,{\mathrm{nm}}$), monolayer graphene, and thin bottom hBN. The latter served as a gate dielectric in the final device configuration (Fig. 1b), and its thickness was determined by atomic force microscopy. The resulting hBN/graphene/hBN stack was then released onto relatively small graphite crystals with thickness of 3–10 nm, which were prepared in advance on an oxidized Si wafer. The stack was large enough to extend outside the bottom graphite region, which allowed us to make quasi-one-dimensional contacts to graphene^31^ without electrically contacting the graphite gate. The metallic contacts were defined by electron-beam lithography. We first used a mixture of CHF_3_ and O_2_ to plasma-etch hBN/graphene and expose the required contact regions. This was followed by deposition of $2\,{\mathrm{nm}}$ Cr/$60\,{\mathrm{nm}}$ Au to make Ohmic contacts to graphene. A gold top gate was then fabricated using another round of electron-beam lithography and, also, served as an etching mask for the final etching step to define the Hall bar geometry.

The devices with other metallic gates (Bi_2_Sr_2_CaCu_2_O_8+*x*_ and TaS_2_) required fabrication in an oxygen- and moisture-free atmosphere of a glovebox^32^ to avoid deterioration of the metal surfaces. Even using glovebox encapsulation, we observed a notable reduction in graphene’s quality for the above gate materials, presumably because of electrical charges at the exposed surfaces (for small $d$, typical $\mu$ became $< 10^5\,{\mathrm{cm}}^2\,{\mathrm{V}}^{ - 1}{\mathrm{s}}^{ - 1}$ and charge inhomogeneity near the NP considerably increased). Accordingly, reliable measurements of $\ell _{{\mathrm{ee}}}$ in this case were only possible at high $n \,{\gtrsim}\, 2.0 \times 10^{12}\,{\mathrm{cm}}^{ - 2}$ (Supplementary Note 2). We also note that encapsulated graphene devices with the conventional gates made by metal deposition on top of a thin gate dielectric ($d \,{<}\, 2\,{\mathrm{nm}}$) exhibited extremely low $\mu$ of only ${\sim} 10^4\,{\mathrm{cm}}^2\,{\mathrm{V}}^{ - 1}{\mathrm{s}}^{ - 1}.$ Such poor electronic quality made it impossible to carry out the $\ell _{{\mathrm{ee}}}$ measurements described in the main text.

### Electrical measurements

The devices were measured in a variable temperature insert that allowed stable $T$ between $2$ and $300\,{\mathrm{K}}$. The standard lock-in amplifier techniques were employed using excitation currents of typically 0.1–1 μA at a frequency of $30.5\,{\mathrm{Hz}}$. For measurements of Hall viscosity, we used the same vicinity geometry as shown in the schematic of Fig. 1a. The distance between injector and detector contacts was usually between $0.5$ and $1.5\,\upmu {\mathrm{m}}$. The viscous Hall resistance was determined as an antisymmetric-in-$B$ component of the vicinity resistance in fields below $\pm 30\,{\mathrm{mT}}$. For the point-contact measurements, we employed the quasi-four-probe geometry by driving the current through the wide contacts (on the left and right in Fig. 1a) and using the leads next to the studied constrictions as voltage probes.

### Proximity screening for systems with the parabolic spectrum

The close-gate condition depends on the density of states at the Fermi energy of the material one wants to control. We have studied graphene not only because of its electronic quality but also because of the low-density of states provided by its Dirac spectrum. For a 2D system with the conventional parabolic spectrum, the close-gate condition is much more difficult to achieve. In the latter case, a conducting gate can provide efficient screening of e–e interactions only for distances $d$ below $\sim\varepsilon \,m_{\mathrm{e}}a_{\mathrm{B}}/(2\,N_{\mathrm{f}}\,m_{{\mathrm{eff}}})$, where $a_{\mathrm{B}} \approx 0.5$ Å is the Bohr radius, $m_{\mathrm{e}}$ and $m_{{\mathrm{eff}}}$ are the free-electron and effective masses, respectively, and $N_{\mathrm{f}}$ is the number of spin/valley flavors. Here, $\varepsilon = \varepsilon _ \bot$ is the perpendicular component of the dielectric permittivity of a gate dielectric. For bilayer graphene^33,34^ with $N_{\mathrm{f}} = 4$, $m_{{\mathrm{eff}}} \ge 0.03\,m_{\mathrm{e}}$ and using hBN as a dielectric ($\varepsilon _ \bot \approx 3.5$), the close-gate condition requires *d* < 7 Å, which is essentially out of experimental reach.

## Supplementary information

Supplementary Information

## Data Availability

The data that support our findings are available upon reasonable request from M.K.

## Code availability

Only commercial software was used in this study.

## References

[1] Yankowitz M (2019). van der Waals heterostructures combining graphene and hexagonal boron nitride. Nat. Rev. Phys..

[2] Ponomarenko LA (2011). Tunable metal-insulator transition in double-layer graphene heterostructures. Nat. Phys..

[3] Lu C-P (2016). Local, global, and nonlinear screening in twisted double-layer graphene. Proc. Natl. Acad. Sci. USA.

[4] Lundeberg MB (2017). Tuning quantum nonlocal effects in graphene plasmonics. Science.

[5] Alcaraz Iranzo D (2018). Probing the ultimate plasmon confinement limits with a van der Waals heterostructure. Science.

[6] Qiu Z (2019). Giant gate-tunable bandgap renormalization and excitonic effects in a 2D semiconductor. Sci. Adv..

[7] Waldecker L (2019). Rigid band shifts in two-dimensional semiconductors through external dielectric screening. Phys. Rev. Lett..

[8] Britnell L (2012). Electron tunneling through ultrathin boron nitride crystalline barriers. Nano Lett..

[9] Imry Y (1997). Introduction to Mesoscopic Physics.

[10] Giuliani GF, Vignale G (2005). Quantum Theory of the Electron Liquid.

[11] Lucas A, Fong KC (2018). Hydrodynamics of electrons in graphene. J. Phys. Condens. Matter.

[12] Bandurin DA (2016). Negative local resistance caused by viscous electron backflow in graphene. Science.

[13] Bandurin DA (2018). Fluidity onset in graphene. Nat. Commun..

[14] Krishna Kumar R (2017). Superballistic flow of viscous electron fluid through graphene constrictions. Nat. Phys..

[15] Berdyugin AI (2019). Measuring Hall viscosity of graphene’s electron fluid. Science.

[16] Principi A, Vignale G, Carrega M, Polini M (2016). Bulk and shear viscosities of the two-dimensional electron liquid in a doped graphene sheet. Phys. Rev. B.

[17] Pellegrino FMD, Torre I, Polini M (2017). Nonlocal transport and the Hall viscosity of two-dimensional hydrodynamic electron liquids. Phys. Rev. B.

[18] Guo H, Ilseven E, Falkovich G, Levitov LS (2017). Higher-than-ballistic conduction of viscous electron flows. Proc. Natl Acad. Sci. USA.

[19] Wallbank JR (2019). Excess resistivity in graphene superlattices caused by umklapp electron–electron scattering. Nat. Phys..

[20] Torre I, Tomadin A, Geim AK, Polini M (2015). Nonlocal transport and the hydrodynamic shear viscosity in graphene. Phys. Rev. B.

[21] Li Q, Das Sarma S (2013). Finite temperature inelastic mean free path and quasiparticle lifetime in graphene. Phys. Rev. B.

[22] Polini, M. & Vignale, G. in *No-nonsense Physicist: An Overview of Gabriele Giuliani’s Work and Life* (eds. Polini, M. et al.) 107–124 (Scuola Normale Superiore, 2016).

[23] Krishna Kumar R (2017). High-temperature quantum oscillations caused by recurring Bloch states in graphene superlattices. Science.

[24] Yankowitz M (2012). Emergence of superlattice Dirac points in graphene on hexagonal boron nitride. Nat. Phys..

[25] Ponomarenko LA (2013). Cloning of Dirac fermions in graphene superlattices. Nature.

[26] Dean CR (2013). Hofstadter’s butterfly and the fractal quantum Hall effect in moiré superlattices. Nature.

[27] Hunt B (2013). Massive Dirac fermions and Hofstadter butterfly in a van der Waals heterostructure. Science.

[28] Cao Y (2018). Unconventional superconductivity in magic-angle graphene superlattices. Nature.

[29] Pizarro JM, Rösner M, Thomale R, Valentí R, Wehling TO (2019). Internal screening and dielectric engineering in magic-angle twisted bilayer graphene. Phys. Rev. B.

[30] Goodwin ZAH (2020). Critical role of device geometry for the phase diagram of twisted bilayer graphene. Phys. Rev. B.

[31] Wang L (2013). One-dimensional electrical contact to a two-dimensional material. Science.

[32] Cao Y (2015). Quality heterostructures from two-dimensional crystals unstable in air by their assembly in inert atmosphere. Nano Lett..

[33] Hwang EH, Das Sarma S (2008). Screening, Kohn anomaly, Friedel oscillation, and RKKY interaction in bilayer graphene. Phys. Rev. Lett..

[34] Borghi G, Polini M, Asgari R, MacDonald AH (2009). Dynamical response functions and collective modes of bilayer graphene. Phys. Rev. B.

