# Publisher Correction: Control of electron–electron interaction in graphene by proximity screening

**DOI:** 10.1038/s41467-020-16708-5

**Published:** 2020-06-11

**Authors:** M. Kim, S. G. Xu, A. I. Berdyugin, A. Principi, S. Slizovskiy, N. Xin, P. Kumaravadivel, W. Kuang, M. Hamer, R. Krishna Kumar, R. V. Gorbachev, K. Watanabe, T. Taniguchi, I. V. Grigorieva, V. I. Fal’ko, M. Polini, A. K. Geim

**Affiliations:** 1 School of Physics and Astronomy, University of Manchester, Manchester, M13 9PL UK; 2 National Graphene Institute, University of Manchester, Manchester, M13 9PL UK; 3 Saint-Petersburg INP, Gatchina, 188300 Russia; 4 National Institute for Materials Science, Tsukuba, 305-0044 Japan; 5 Dipartimento di Fisica dell’Università di Pisa, Largo Bruno Pontecorvo 3, 56127 Pisa, Italy; 6 Istituto Italiano di Tecnologia, Graphene Labs, Via Morego 30, 16163 Genova, Italy

**Keywords:** Nanoscience and technology, Physics

Correction to: *Nature Communications* 10.1038/s41467-020-15829-1, published online 11 May 2020.

The original version of this Article contained an error in the title which incorrectly reads ‘Control of electron–electron interaction in graphene by proximity screenings’. The correct version is ‘*Control of electron–electron interaction in graphene by proximity screening*’. This has been corrected in both the PDF and HTML versions of the Article.

