# Supplementary material for: Control of electron-electron interaction in graphene by proximity screening
Source: Nat Commun. 2020 May 11;11:2339. doi: 10.1038/s41467-020-15829-1 (PMC7214472; doi:10.1038/s41467-020-15829-1)
Supplement: Supplementary file 1 — Supplementary Information [file 41467_2020_15829_MOESM1_ESM.pdf]

SUPPLEMENTARY INFORMATION

Control of electron-electron interaction in graphene by proximity screening

M. Kim *et al.*

Supplementary Note 1

Mean free path and mobility. We carefully examined transport characteristics for several monolayer graphene devices with different dielectric thicknesses d. The mean free path ℓ with respect to momentum-non-conserving collisions was determined from the measured longitudinal resistivity ρ by using the Drude formula. The carrier density n was found from Hall measurements. Typical results for ℓ as a function of n are shown in Supplementary Fig. 1a. The mean free path first increases with increasing n and then saturates for $n \gtrsim 1.0 \times 10^{12}$ cm^{-2}. It monotonically decreases with temperature T as expected. Such behavior was observed for all the measured devices independently of their d. This is elucidated by Supplementary Fig. 1b that shows ℓ for different d at the given n at room T. One can see that the measured ℓ varied only slightly, from ~ 0.7 to $1.1\ \mu\text{m}$, depending on graphene device's quality. Similarly, carrier mobilities $\mu(n)$ exhibited little dependence on d (Supplementary Fig. 1c).

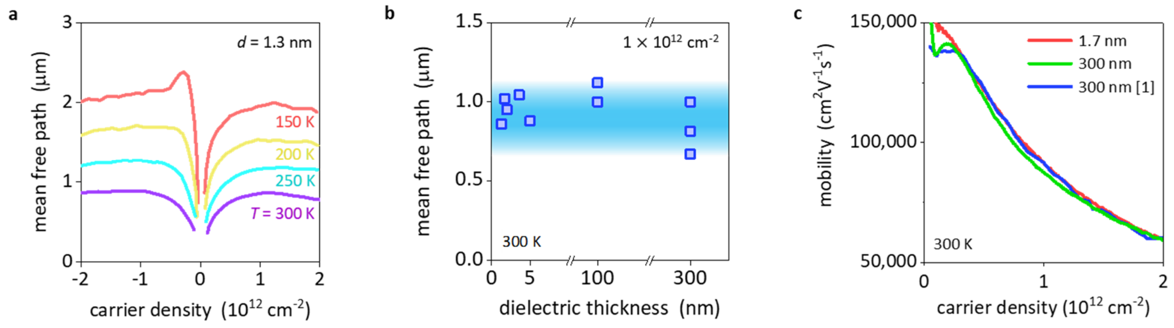

Supplementary Figure 1 | Transport characteristics for different thicknesses of the gate dielectric. a, $\ell(n)$ for a graphene device with $d \approx 1.3$ nm at a few representative T. **b,** ℓ for devices with different d at 300 K; $n = 1 \times 10^{12}\ \text{cm}^{-2}$. **c,** Density dependence $\mu(n)$ at room T. The mobilities measured for devices with different d collapse on a single curve. The red and green curves are for gate dielectrics with ≈ 1.7 and 300 nm, respectively. The blue curve: Data from ref. 1 to indicate the generality of such behavior at elevated T.

Supplementary Note 2

Different screening materials. Because graphite is a semimetal[2,3] with a relatively low carrier concentration of the order of $10^{19}\ \text{cm}^{-3}$, we have checked the generality of our conclusions using other metallic substrates, namely $Bi_2Sr_2CaCu_2O_{8+x}$ (BSCCO) and TaS_2 which have concentrations of $\sim 10^{22}\ \text{cm}^{-3}$ (ref. 4). To this end, devices similar to those shown in Fig. 1a of the main text were fabricated but, instead of graphite, cleaved BSCCO and TaS_2 crystals served as metallic substrates. To protect them from degradation, fabrication had to be carried out in an argon atmosphere of a glovebox as discussed in Methods. The carrier mobility μ for the latter devices was comparable to that of the devices made with graphite screening gates but only for high $n \gtrsim 2 \times 10^{12}\ \text{cm}^{-2}$. At lower n, the electronic quality was insufficient to probe electron viscosity because of short ℓ, presumably due to extra charges that appear on the metallic surfaces exposed to the ambient atmosphere. Accordingly, for the alternative screening substrates, we worked in the high n regime to measure the viscous Hall resistance and then extract ℓ_{ee}. Supplementary Fig. 2 shows the resulting ℓ_{ee} for graphene devices

using various screening materials. Within our experimental accuracy, no difference in ℓ_{ee} could be noticed, and the experimental data closely followed the theoretical predictions.

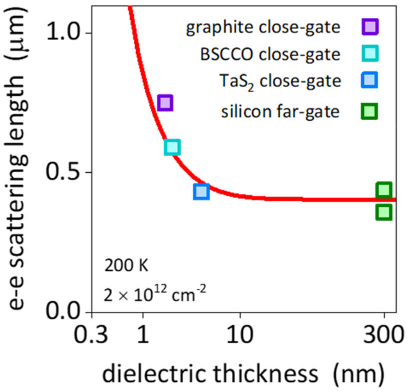

Supplementary Figure 2 | Electron-electron scattering in devices with different materials used for proximity screening. Symbols: Measured ℓ_{ee} at $200\ \mathrm{K}$ and $2 \times 10^{12}\ \mathrm{cm}^{-2}$ (color coded). Solid curve: Theory.

Supplementary Note 3

Point contact geometry. For completeness, we also measured ℓ_{ee} using the point-contact geometry[5]. By applying an electric current through a graphene constriction and monitoring a voltage drop at nearby contacts (see Fig. 1a of the main text), the point contact resistance R_{PC} was measured. Supplementary Fig. 3a shows $R_{\mathrm{PC}}(T)$ for a graphene constriction with a geometrical width of $\sim 350\ \mathrm{nm}$ as found by atomic force microscopy. The transport width w of the constriction was somewhat smaller, $\sim 270\ \mathrm{nm}$, as found by fitting $R_{\mathrm{PC}}(n)$ at liquid-helium T by the standard Sharvin formula ($R_{\mathrm{Sh}} = \frac{\pi h}{4e^2} \frac{1}{w\sqrt{n\pi}}$). The smaller width inferred from the fit is expected and presumably caused by edge roughness[5]. R_{PC} exhibited a nonmonotonic T dependence, becoming at intermediate T notably smaller than the ideal value in the ballistic limit (Supplementary Fig. 3a). This "superballistic" behavior is due to e-e scattering as discussed elsewhere[5,6].

To extract ℓ_{ee} from the measurements such as those shown in Supplementary Fig. 3a, we used the expression[5,6]

$$R_{\mathrm{PC}} = (1/R_{\mathrm{Sh}} + G_v)^{-1} + R_{\mathrm{C}}$$

where $R_{\mathrm{C}} = b\rho$ is the contact resistance arising from the wide regions near the point contact. R_{C} can be determined accurately for the known ρ whereas the dimensionless coefficient b is found from numerical simulations[5]. The viscous contribution G_v to the point-contact conductivity is given by[6] $G_v = \frac{\sqrt{|n|\pi} e^2 w^2}{8\hbar \ell_{ee}}$. Supplementary Fig. 3b shows examples of $\ell_{ee}(T)$ found using the above analysis. The behavior of ℓ_{ee} agrees well with that found from the Hall viscosity measurements in the main text. For example, ℓ_{ee} is clearly enhanced for devices with close gates. The experimental data also agree with theory whereas relatively small deviations from it at high T are due to non-Fermi-liquid corrections as reported in ref. 5 and, also, explained below.

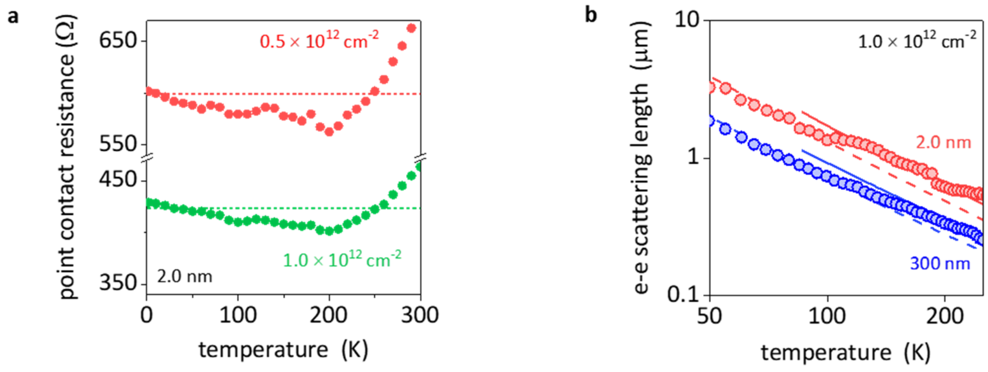

Supplementary Figure 3 | Electron-electron scattering length found from point-contact measurements. a, Point-contact resistance for a device with a close gate ($d \approx 2.0$ nm) at different n (color coded). Dots: Experimental data. The dashed lines indicate the ideal value expected in the ballistic limit at low T. **b,** $\ell_{\rm ee}(T)$ for $d = 2.0$ (red) and 300 nm (blue) for the given n. Symbols: Experiment. Dashed curves: Theoretical predictions with no fitting parameters. Solid curves: Same theory data but multiplied by a numerical coefficient of 1.3.

Supplementary Note 4

Microscopic theory of screened electron-electron scattering. In this Note we briefly described our approach to calculate $\ell_{\rm ee} = v_{\rm F}\tau_{\rm ee}$. The mean free time $\tau_{\rm ee}$ for e-e scattering is controlled by the one-body Green's function $G_\lambda(\boldsymbol{k},\omega)$, where $\lambda = \pm 1$ is a band index ($\lambda = +1$ for conduction-band states and $\lambda = -1$ for valence-band states). This quantity satisfies the Dyson equation (setting $\hbar = 1$), $G_\lambda(\boldsymbol{k},\omega) = [\omega - \xi_{\boldsymbol{k},\lambda} - \Sigma_\lambda(\boldsymbol{k},\omega)]^{-1}$, where $\xi_{\boldsymbol{k},\lambda}$ are single-particle band energies measured from the chemical potential μ and $\Sigma_\lambda(\boldsymbol{k},\omega)$ is the retarded self-energy. The latter quantity needs to be approximated. In weakly-correlated materials, a good approximation is the so-called GW approximation[7,8] in which the electron self-energy is expanded to first order in the dynamically screened Coulomb interaction $W(\boldsymbol{q}, i\Omega)$

$$\Sigma_\lambda(\boldsymbol{k}, i\omega_n) = -k_{\rm B}T \sum_{\lambda'=\pm 1} \int \frac{d^2\boldsymbol{q}}{(2\pi)^2} \sum_{m=-\infty}^{+\infty} W(\boldsymbol{q}, i\Omega_m) F_{\lambda\lambda'}(\theta_{\boldsymbol{k},\boldsymbol{k}-\boldsymbol{q}}) G_{\lambda'}(\boldsymbol{k}-\boldsymbol{q}, i\omega_n + i\Omega_m) \quad (1)$$

where $\omega_n = (2n+1)\pi k_B T$ is a fermionic Matsubara frequency, the sum runs over all the bosonic Matsubara frequencies $\Omega_m = 2m\pi k_{\rm B}T$, $\theta_{\boldsymbol{k},\boldsymbol{k}-\boldsymbol{q}}$ is the angle between $\boldsymbol{k}$ and $\boldsymbol{k}-\boldsymbol{q}$, and $F_{\lambda\lambda'}(\varphi) = [1 + \lambda\lambda'\cos(\varphi)]/2$ is the so-called chirality factor[9]. The retarded self-energy can be obtained after analytical continuation $i\omega_n \to \omega + i0^+$. For the sake of concreteness and without loss of generality due to particle-hole symmetry, we focus on electron-doped graphene, i.e. on the case $E_{\rm F} > 0$, where $E_{\rm F} = v_{\rm F}k_{\rm F}$ is the Fermi energy. Here, $v_{\rm F} \sim 10^6$ m/s ($k_{\rm F} = \sqrt{\pi n}$) is the Fermi velocity (Fermi wave number), with $n > 0$ the electron density.

The Dyson equation combined with the approximate GW expression for the electron self-energy define a *self-consistent* approximation, whose self-energy and Green's function can be calculated based on an iterative procedure. One first calculates the self-energy from the GW expression by using in the right-hand side of the *non-interacting* Green's function $G_{\lambda'}(\boldsymbol{k}-\boldsymbol{q}, i\omega_n + i\Omega_m) \to G^{(0)}_{\lambda'}(\boldsymbol{k}-\boldsymbol{q}, i\omega_n + i\Omega_m) = 1/(i\omega_n - \xi_{\boldsymbol{k}-\boldsymbol{q},\lambda'})$. The obtained result is then replaced in the right-hand side of the

Dyson equation, obtaining a new Green's function. The latter is then used to re-calculate the self-energy via the GW equation, until self-consistency is achieved. Now, the key point is that deep in the Fermi liquid regime, i.e. for $|\boldsymbol{k}| \simeq k_{\rm F}$ and $|\omega|/E_{\rm F}, k_{\rm B}T/E_{\rm F} \ll 1$, the self-energy is a small correction to the bare band energy $\xi_{\boldsymbol{k},\lambda}$ and such self-consistency is unnecessary. In this limit indeed, quasiparticles are long lived because of the ineffectiveness of e-e collisions (Pauli blocking) and $\mathrm{Im}[\Sigma_+(\boldsymbol{k},\omega)] \propto (k_{\rm B}T/E_{\rm F})^2 + (\omega/E_{\rm F})^2$, modulo logarithmic corrections. In this regime, it is therefore well justified to replace $G_{\lambda'}(\boldsymbol{k}-\boldsymbol{q}, i\omega_n + i\Omega_m)$ with $G^{(0)}_{\lambda'}(\boldsymbol{k}-\boldsymbol{q}, i\omega_n + i\Omega_m)$ in the right-hand side of the GW equation obtaining the so-called $G^{(0)}W$ approximation[7,8].

Since this is the simplest possible theory, we use the $G^{(0)}W$ approximation also away from the Fermi liquid regime, being aware of the fact, however, that the lack of full self-consistency is expected to lead to inaccuracies. In particular, it is easy to demonstrate that $\ell_{\rm ee}|_{G^{(0)}W} < \ell_{\rm ee}|_{GW}$. Since in weakly correlated materials such as graphene the GW approximation is expected to be quantitatively good (i.e. $\ell_{\rm ee}|_{GW}$ is expected to be close to the experimentally value of $\ell_{\rm ee}$), we do expect the non-self-consistent result $\ell_{\rm ee}|_{G^{(0)}W}$ to systematically *underestimate* the experimentally measured $\ell_{\rm ee}$. Therefore, in the main text, we have compared experimental data with $\ell_{\rm ee}|_{G^{(0)}W}$ after multiplying the latter by a *constant* enhancement factor of 1.3, which is independent of all microscopic parameters (Fig. 2 of the main text).

The quantity $\ell_{\rm ee}|_{G^{(0)}W}$ can be calculated numerically once one specifies the dynamically screened potential $W(\boldsymbol{q}, i\Omega_m)$. In the random phase approximation[7], $W(\boldsymbol{q},\omega) = V_{\boldsymbol{q}}/[1 - V_q\chi^{(0)}_{nn}(\boldsymbol{q},\omega)]$, where $\chi^{(0)}_{nn}(\boldsymbol{q},\omega)$ is the well-known density-density response function of doped graphene[9] and $V_{\boldsymbol{q}}$ is the 2D Fourier transform of the e-e interaction potential, which is sensitive to screening caused by nearby gates and gate dielectrics. For our conductor/hBN/graphene/hBN/conductor heterostructures, electrostatic calculations yield

$$V_{\boldsymbol{q}} = \frac{4\pi e^2}{q\sqrt{\epsilon_x\epsilon_z}} \frac{\sinh\left(qd\sqrt{\frac{\epsilon_x}{\epsilon_z}}\right)\sinh\left(qd'\sqrt{\frac{\epsilon_x}{\epsilon_z}}\right)}{\sinh\left[q(d+d')\sqrt{\frac{\epsilon_x}{\epsilon_z}}\right]} \quad (2)$$

where d' (d) is the thickness of hBN above (below) graphene, and ϵ_x and ϵ_z are the static in-plane and out-of-plane permittivities of hBN. Two gates, modelled as perfect conductors, are placed above and below graphene at distances d' and $d \ll d'$, respectively, and are separated from graphene by hBN. Numerical calculations of $\ell_{\rm ee}|_{G^{(0)}W}$ have been carried out by using this effective screened e-e interaction for sufficiently large $d' \approx 60$ nm and known $\epsilon_x = 6.70$, and $\epsilon_z = 3.56$ (see, for example, ref. 10). Values of d, n, and T were variables in our calculations. Pertinent results are presented in Fig. 2 of the main text.

For a qualitative understanding of the role of screening, it is useful to obtain an approximate expression for $\ell_{\rm ee}|_{G^{(0)}W}$ as a function of all system parameters. To this end, we follow ref. 8 and derive a formula for $\ell_{\rm ee}|_{G^{(0)}W}$ which is exact in the Fermi-liquid regime, $k_{\rm B}T \ll E_{\rm F}$. The calculations follow essentially the same steps as in ref. 8, modulo minor differences, which stem from the regularity of $V_{\boldsymbol{q}}$ in the long-wavelength $q \to 0$ limit and will be discussed elsewhere. Indeed, $\lim_{q\to 0} V_{\boldsymbol{q}} = 4\pi e^2 d_{\rm eff}/\epsilon_z \equiv V_0$, where $d_{\rm eff} = dd'/(d+d')$. This formula allows a simple interpretation. Having the two, top and

bottom, gates is like having two capacitors in parallel. Indeed, we can write $V_0 = e^2/C_{\rm eff}$, where the $C_{\rm eff} = C_d + C_{d'}$ is the sum of the two relevant geometrical capacitances (per unit area), $C_d = \epsilon_z/(4\pi d)$ and $C_d = \epsilon_z/(4\pi d')$. After restoring $\hbar$, we obtain

$$\lim_{\frac{k_{\rm B}T}{E_{\rm F}}\to 0} \ell_{\rm ee}|_{G^{(0)}W} = \frac{4\hbar v_{\rm F}E_{\rm F}}{\pi}\frac{1}{(k_{\rm B}T)^2\ln\left(\frac{2E_{\rm F}}{k_{\rm B}T}\right)}\left(\frac{1+2d_{\rm eff}q_{\rm TF}}{2d_{\rm eff}q_{\rm TF}}\right)^2 \quad (3).$$

Eq. 2 in the main text is simply obtained from Supplementary Eq. 3 by taking the limit $d' \to \infty$.

Before concluding this Note, let us comment on possible corrections to our model caused by the fact that real gates are not the assumed perfect conductors. The effect of a finite density-of-states can be estimated using the Thomas-Fermi approximation. It is possible to show that, in this approximation, the previous asymptotic result for $\ell_{\rm ee}|_{G^{(0)}W}$ in the limit $k_{\rm B}T \ll E_{\rm F}$ holds if one replaces $d \to d + 1/q_{\rm TF}$, where $q_{\rm TF}$ is the Thomas-Fermi screening wavenumber in gate's material. The screening length $1/q_{\rm TF}$ of graphite is theoretically calculated to be 5 to 7 Å, taking into account the existence of an intra-layer charge polarization and inter-layer electron tunneling[11,12]. For our other metallic substrates, we find $1/q_{\rm TF} \approx$ 2 Å by employing a Thomas-Fermi screening model for a 3D conductor, which corresponds to interatomic distances as expected.

Supplementary Note 5

Suppression of umklapp e-e scattering by proximity screening. It has been shown[13] that umklapp e-e scattering ($U_{\rm ee}$) substantially increases the resistivity of high-quality graphene-on-hBN superlattices (SL) in the range of T between 50 and 200 K. The SL potential is generated by the moiré pattern that has a period $\lambda \approx 15$ nm for a perfectly aligned graphene and hBN crystals. $U_{\rm ee}$ is a process where a crystal lattice (superlattice in our case) provides interacting electrons with an additional momentum kick such that the momentum conservation takes the form $\mathbf{k_3} + \mathbf{k_4} = \mathbf{k_1} + \mathbf{k_2} + \mathbf{g}$, where $\mathbf{k}_{1,2}$ and $\mathbf{k}_{3,4}$ are the initial and final momenta of two electrons near the Fermi level, and $\mathbf{g} = (\mathbf{g}_x, \mathbf{g}_y)$ is a reciprocal vector of the crystal (Supplementary Fig. 4a). Such a process becomes possible only for $4k_{\rm F} > g$, where $g = |\mathbf{g}| = \frac{4\pi}{\sqrt{3}\lambda}$ is the length of one of the 6 shortest vectors of the reciprocal SL.
The contribution of $U_{\rm ee}$ towards graphene's resistivity ρ is given by[13]

$$\Delta\rho = \frac{\hbar\pi}{e^2k_{\rm F}}\, l_{U{\rm ee}}^{-1} \text{ with } l_{U{\rm ee}}^{-1} = \frac{(k_{\rm B}T)^2}{12\,\pi^2 v_{\rm F}^4 k_{\rm F}}\sum_{\rm g}({\rm g}_x)^2\int\frac{d\theta_{{\rm k}_1}d\theta_{{\rm k}_3}}{\left|\sin\left(\theta_{{\rm k}_2}-\theta_{{\rm k}_4}\right)\right|}\left|\sum_{i={\rm I}}^{\rm IV}\sum_{s'=\pm}M_{ss'}^{(i)}\right|^2 \quad (4)$$

where $\theta_{\mathbf{k}}$ denotes an angle between $\mathbf{k}$ and x-axis, $s = \pm$ stands for the conductance/valence-band states (fixed by doping), and s' marks virtual intermediate states. In Supplementary Eq. 4, the inverse umklapp scattering length, $l_{U{\rm ee}}^{-1}$, is determined by the sum of four Feynman diagrams shown in Supplementary Fig. 4b, each described by the scattering amplitude $M_{ss'}^{(i)}$ $(i = {\rm I, II, III, IV})$. For example, the first diagram gives a contribution

$$M_{ss'}^{\rm (I)} = \frac{W(\mathbf{g})\frac{1+ss'e^{i\theta_{{\rm k}_1+{\rm g}}-i\theta_{{\rm k}_3}}}{2}V(|\mathbf{k}_2-\mathbf{k}_4|)\frac{1+e^{i\theta_{{\rm k}_2}-i\theta_{{\rm k}_4}}}{2}}{sv|\mathbf{k}_1|-s'v|\mathbf{k}_1+\mathbf{g}|} \quad (5)$$

where $W(\mathbf{g})$ stands for the scattering amplitude of an electron off the moiré SL[14,15], and

$$V(q) = \frac{V_q(q,d,d')}{1+V_q(q,d,d')\Pi(q)} \tag{6}$$

is the Coulomb interaction screened by both gate and the Fermi sea in graphene; $\Pi(q \le 2k_{\rm F}) = \frac{2k_{\rm F}}{\hbar\pi v_{\rm F}}$ is the Thomas-Fermi polarization operator[16-20]. From the form of $V_{\boldsymbol{q}}$ in Supplementary Eq. 2, it is straightforward to see that, for e-e scattering with the momentum transfer $q\sim g/2$, the gate starts playing a notable screening role only if $d_{\rm eff} \lesssim \sqrt{\frac{\epsilon_z}{\epsilon_x}}\frac{1}{g} \approx 0.1\lambda$ ($d_{\rm eff} \approx d \ll d'$). Expressions for the other diagrams in Supplementary Fig. 4b can be obtained by changing input momenta and $\boldsymbol{q}$ in Supplementary Eq. 5.

The $U_{\rm ee}$ contribution, computed using the same SL parameters as those in refs. 13 and 21, exhibits a significant suppression for $d \lesssim 2$ nm (Supplementary Fig. 4c). In these calculations, the absolute value of $\Delta\rho \propto l_{U{\rm ee}}^{-1}$ obviously depends on the moiré potential's strength. To compare the effect of proximity screening on $U_{\rm ee}$, without relying on a detailed choice of SL parameters, we also plot the ratio $l_{U{\rm ee}}^{-1}(\infty)/l_{U{\rm ee}}^{-1}(d)$ at $n \approx -\frac{1}{2}n_0$ and compare the theoretical results with the experimentally found ratio $\Delta\rho(\infty)/\Delta\rho(d)$ [see Fig. 3 of the main text].

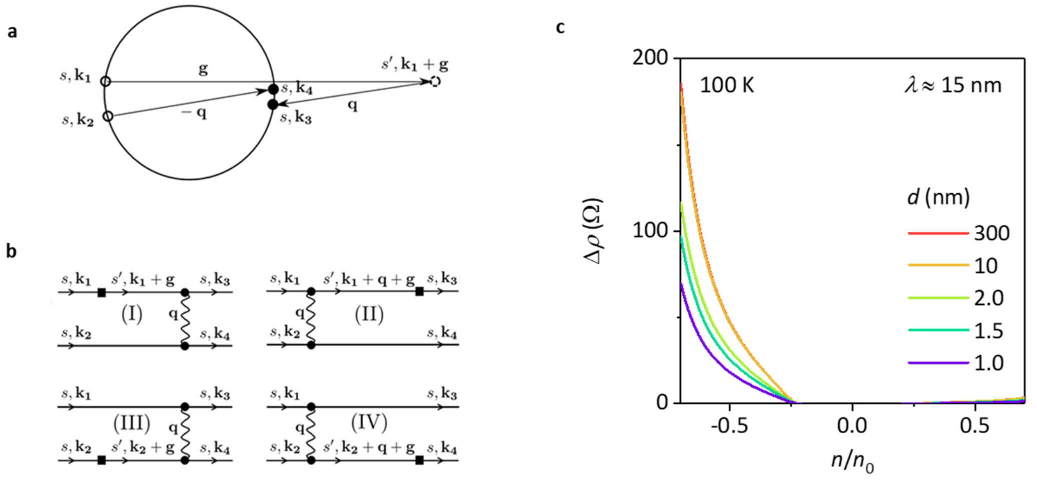

Supplementary Figure 4 | Screened umklapp e-e scattering in graphene superlattices. a, Kinematics of $U_{\rm ee}$ scattering. **b,** Feynman diagrams for $M_{ss'}^{(i)}$. **c,** Additional resistivity caused by $U_{\rm ee}$ for different distances to the gate (color coded).

Supplementary References

1 Wang, L. *et al.* One-dimensional electrical contact to a two-dimensional material. *Science* **342**, 614-617 (2013).

2 Dresselhaus, M. S. & Dresselhaus, G. Intercalation compounds of graphite. *Adv. Phys.* **51**, 1-186 (2002).

3 Yin, J. *et al.* Dimensional reduction, quantum Hall effect and layer parity in graphite films. *Nat. Phys.* **15**, 437-442 (2019).

4 Sterpetti, E., Biscaras, J., Erb, A. & Shukla, A. Comprehensive phase diagram of two-dimensional space charge doped $Bi_2Sr_2CaCu_2O_{8+x}$. *Nat. Commun.* **8**, 2060 (2017).

5 Krishna Kumar, R. *et al.* Superballistic flow of viscous electron fluid through graphene constrictions. *Nat. Phys.* **13**, 1182-1185 (2017).

6 Guo, H., Ilseven, E., Falkovich, G. & Levitov, L. S. Higher-than-ballistic conduction of viscous electron flows. *PNAS* **114**, 3068-3073 (2017).

7 Giuliani, G. F. & Vignale, G. *Quantum theory of the electron liquid*. (Cambridge University Press, Cambridge, 2005).

8 Polini, M. & Vignale, G. in *No-nonsense Physicist: An overview of Gabriele Giuliani's work and life* (eds Marco Polini, Giovanni Vignale, Vittorio Pellegrini, & Jainendra K. Jain) 107-124 (Scuola Normale Superiore, 2016).

9 Kotov, V. N., Uchoa, B., Pereira, V. M., Guinea, F. & Castro Neto, A. H. Electron-electron interactions in graphene: current status and perspectives. *Rev. Mod. Phys.* **84**, 1067-1125 (2012).

10 Woessner, A. *et al.* Highly confined low-loss plasmons in graphene–boron nitride heterostructures. *Nat. Mater.* **14**, 421 (2014).

11 Visscher, P. B. & Falicov, L. M. Dielectric screening in a layered electron gas. *Phys. Rev. B* **3**, 2541-2547 (1971).

12 Guinea, F. Charge distribution and screening in layered graphene systems. *Phys. Rev. B* **75**, 235433 (2007).

13 Wallbank, J. R. *et al.* Excess resistivity in graphene superlattices caused by umklapp electron–electron scattering. *Nat. Phys.* **15**, 32-36 (2019).

14 Wallbank, J. R., Patel, A. A., Mucha-Kruczyński, M., Geim, A. K. & Fal'ko, V. I. Generic miniband structure of graphene on a hexagonal substrate. *Phys. Rev. B* **87**, 245408 (2013).

15 Wallbank, J. R., Mucha-Kruczyński, M., Chen, X. & Fal'ko, V. I. Moiré superlattice effects in graphene/boron-nitride van der Waals heterostructures. *Ann. Phys.* **527**, 359-376 (2015).

16 Gorbar, E. V., Gusynin, V. P., Miransky, V. A. & Shovkovy, I. A. Magnetic field driven metal-insulator phase transition in planar systems. *Phys. Rev. B* **66**, 045108 (2002).

17 Ando, T. Screening effect and impurity scattering in monolayer graphene. *J. Phys. Soc. Jpn.* **75**, 074716 (2006).

18 Wunsch, B., Stauber, T., Sols, F. & Guinea, F. Dynamical polarization of graphene at finite doping. *New J. Phys.* **8**, 318-318 (2006).

19 Hwang, E. H. & Das Sarma, S. Dielectric function, screening, and plasmons in two-dimensional graphene. *Phys. Rev. B* **75**, 205418 (2007).

20 Ong, Z.-Y. & Fischetti, M. V. Charged impurity scattering in top-gated graphene nanostructures. *Phys. Rev.* B **86**, 121409 (2012).

21 Lee, M. *et al.* Ballistic miniband conduction in a graphene superlattice. *Science* **353**, 1526-1529 (2016).
